# Padlet: a tool for fostering collaborative learning and feedback literacy in dental education

**DOI:** 10.3389/fmed.2024.1357068

**Published:** 2025-01-22

**Authors:** Avita Rath

**Affiliations:** Faculty of Dentistry, SEGi University, Kota Damansara, Malaysia

**Keywords:** Padlet online, collaborative learning, feedback literacy, undergraduate dental education, formative assessment, trust, Nicol and MacFarlane's feedback principles, digital learning platforms

## Abstract

**Background:**

While health professions education has embraced collaborative and co-creative learning approaches, the integration of digital technologies into feedback practices remains limited, especially in undergraduate dental education. This study investigated the impact of Padlet, a digital pinboard, on collaborative learning and feedback literacy among undergraduate dental students during a formative assessment activity guided by Nicol and MacFarlane's feedback principles.

**Methods:**

A convenience sample of 39 Year 3 dental students (25 women and 14 men, mean age = 22) enrolled in a Bachelor of Dental Surgery program at a private dental school in Malaysia participated in a week-long Padlet-based peer feedback activity focused on periodontics. Thematic analysis was conducted on student interactions and reflections collected from Padlet posts and individual student reflections.

**Results:**

The study yielded promising outcomes. Padlet's asynchronous and anonymous nature fostered in-depth discussions, broader participation, and constructive feedback. Students reported a boost in confidence, increased engagement, and a sense of camaraderie. Thematic analysis revealed the successful application of Nicol and MacFarlane's feedback principles, including clarifying expectations, promoting self-assessment, facilitating teacher–student dialogue, and encouraging reflection and action.

**Conclusion:**

The findings of this study suggest that Padlet, with its unique features, offers a valuable tool for educators seeking to foster collaborative learning and feedback literacy in dental education. Padlet's affordances can significantly enhance collaborative learning and feedback literacy, promoting a trusting environment for student-centered learning and self-regulation in dental education. The study's findings suggest that Padlet can foster cognitive flexibility, allowing students to consider multiple perspectives and adapt their thinking. The platform's asynchronous nature and anonymity feature appeared to contribute to a sense of community and psychological safety, fostering trust among students. The findings of this study have practical implications for educators seeking to implement effective feedback practices and leverage technology to create engaging learning experiences that foster trust, collaboration, and, ultimately, student success.

## Introduction

Health professions education has shifted from traditional, teacher-centric feedback practices toward more collaborative and co-creative approaches, embracing the versatility of digital technologies to enhance feedback (1). However, integrating these technologies into feedback practices, particularly in undergraduate dental education, remains limited, especially in Malaysia (2).

Formative feedback, defined as “information communicated to learners during the learning process to help them identify strengths and weaknesses and improve their performance” (3), is crucial in dental education for fostering self-regulated learning and clinical competence development. In the context of undergraduate dental education at a private Malaysian dental school, feedback is predominantly delivered face-to-face in clinics, occasionally supplemented by synchronous online discussions for complex theoretical topics. While these methods offer some benefits, they may hinder the ongoing dialogue and peer comparison crucial for developing evaluative judgment and feedback literacy—the ability to understand and effectively utilize feedback (4). As Carless and Boud (3) state, “feedback literacy only happens if learners can compare their work with others and make those comparisons explicit” (p. 1318).

The preference of undergraduate students for corrective feedback from academics further complicates the situation. This reliance can be time-consuming for academics, limits conversational learning opportunities (5), and discourages active participation, particularly among shy students. Encouraging “sustained participation” is vital to developing self-regulatory practices (6), critical reflection (7), effective decision-making (8), and interpersonal communication (9)—all sought-after attributes for dental professionals (10).

## Literature review

### Theoretical foundations of collaborative learning and feedback

The present study explores the intersection of collaborative learning, feedback literacy, and trust within the context of dental education, utilizing the digital platform Padlet. To provide a robust theoretical foundation for this investigation, I draw upon several key theoretical frameworks illuminating the complex dynamics at play.

### Social constructivism and collaborative learning

Collaborative learning, a cornerstone of this study, finds its theoretical roots in social constructivism, a perspective that emphasizes the social construction of knowledge through interaction and collaboration (11). Vygotsky's (12) sociocultural theory, a prominent branch of social constructivism, posits that learning is a mediated process that occurs within a zone of proximal development, where learners engage in collaborative activities with more knowledgeable others to achieve shared goals. In this context, Padlet functions as a digital space where students can interact, exchange ideas, and co-construct knowledge, thereby fostering a deeper understanding of the subject matter. The asynchronous nature of Padlet allows for sustained reflection and discourse, enabling students to construct and confirm meaning, which aligns with the concept of cognitive presence within the Community of Inquiry framework (13).

### Feedback literacy and self-regulated learning

Feedback literacy, the ability to understand and effectively utilize feedback, is another critical aspect of student learning (3). It involves not only receiving feedback but also actively seeking it, interpreting it constructively, and using it to improve one's performance (4). The development of feedback literacy is closely linked to self-regulated learning, a process where learners set goals, monitor their progress, and adjust their strategies to achieve desired outcomes (6). According to Nicol (2021), the innate human nature to indulge in comparison dialogue could be leveraged for the development of self-assessment and subsequently, self-regulatory skills, which provides a valuable framework for understanding how Padlet can support the cultivation of feedback literacy (14). By enabling students to compare their work with others, engage in dialogue, and receive timely and constructive feedback, Padlet can empower learners to take ownership of their learning and develop the metacognitive skills necessary for self-regulation.

### Trust and social interdependence

Trust plays a pivotal role in fostering effective collaborative learning and feedback processes (16). In educational settings, trust refers to the belief that others will act in ways that benefit the group and contribute to achieving shared goals (17). Building trust is crucial for successful collaborative learning. Bryk and Schneider (15) identified trust as a core resource for improvement in educational contexts, enabling collaboration, shared accountability, and effective communication among learners. When trust is present, students are more likely to participate actively, share their ideas openly, and provide constructive feedback to their peers (18). Conversely, lack of trust can lead to apprehension, reluctance to engage, and superficial interactions (19). Trust can be cultivated through various mechanisms, including clear communication, mutual respect, shared responsibility, and a sense of belonging within the learning community (20).

The concept of trust is closely intertwined with social interdependence theory, which suggests that collaborative outcomes are greatly influenced by the level of trust and cooperation among group members (7). According to findings from previous research (17, 18), trust has been shown to significantly impact students' willingness to engage in open and constructive dialogue, an essential component of effective feedback and learning. These past findings highlight the importance of psychological safety in educational settings, where power dynamics can often hinder honest communication (22). The anonymity feature of digital platforms such as Padlet, as explored in the present study, could alleviate such anxieties by creating a more equitable and safe environment for students to express their thoughts freely, which is crucial for building trust and promoting transformative learning (23).

### Padlet, connectivism, and the community of inquiry

Padlet's affordances, such as its user-friendliness, intuitive interface, and anonymity features, create a virtual collaborative space that encourages authentic interactions and mitigates evaluation anxiety (23). By enabling students to share their ideas, provide feedback, and engage in dialogue in a safe and supportive environment, Padlet has the potential to foster trust and facilitate the development of collaborative learning and feedback literacy skills.

The use of Padlet also aligns with the principles of connectivism, a learning theory that emphasizes the importance of networks and connections in the digital age (25). Padlet allows students to create and share content, connect with their peers, and access a vast network of information and resources. This networked learning environment can enhance students' understanding of complex topics and promote the development of critical thinking and problem-solving skills (26).

Furthermore, the implementation of Padlet can be seen through the lens of the Community of Inquiry (CoI) framework (13). The CoI model highlights the importance of three core elements in online learning environments: cognitive presence, social presence, and teaching presence. Cognitive presence refers to the extent to which learners are able to construct and confirm meaning through sustained reflection and discourse. Social presence pertains to the ability of learners to project their personal characteristics into the community, thereby establishing interpersonal relationships and emotional connections. Teaching presence encompasses the design, facilitation, and direction of cognitive and social processes for the purpose of realizing personally meaningful and educationally worthwhile learning outcomes. Padlet, with its interactive features and collaborative potential, can facilitate all three presences, creating a rich and engaging online learning experience (13, 18, 27).

## Methods

This study employed a qualitative case study design to explore the experiences of 39 Year 3 dental students using Padlet for a formative peer feedback activity focused on periodontics topics. The participants included 25 women and 14 men, with an average age of 22. All students were enrolled in their third year of the Bachelor of Dental Surgery program at a private dental school in Malaysia.

Ethical approval for the study was obtained from the SEGi University Research Ethics Committee (ethics no. SEGIEC/SR/FOD/131/2023-24). Informed and written consent was obtained from all participants prior to their involvement in the study. Participation was entirely voluntary.

### Padlet activity

**Figure 1:**
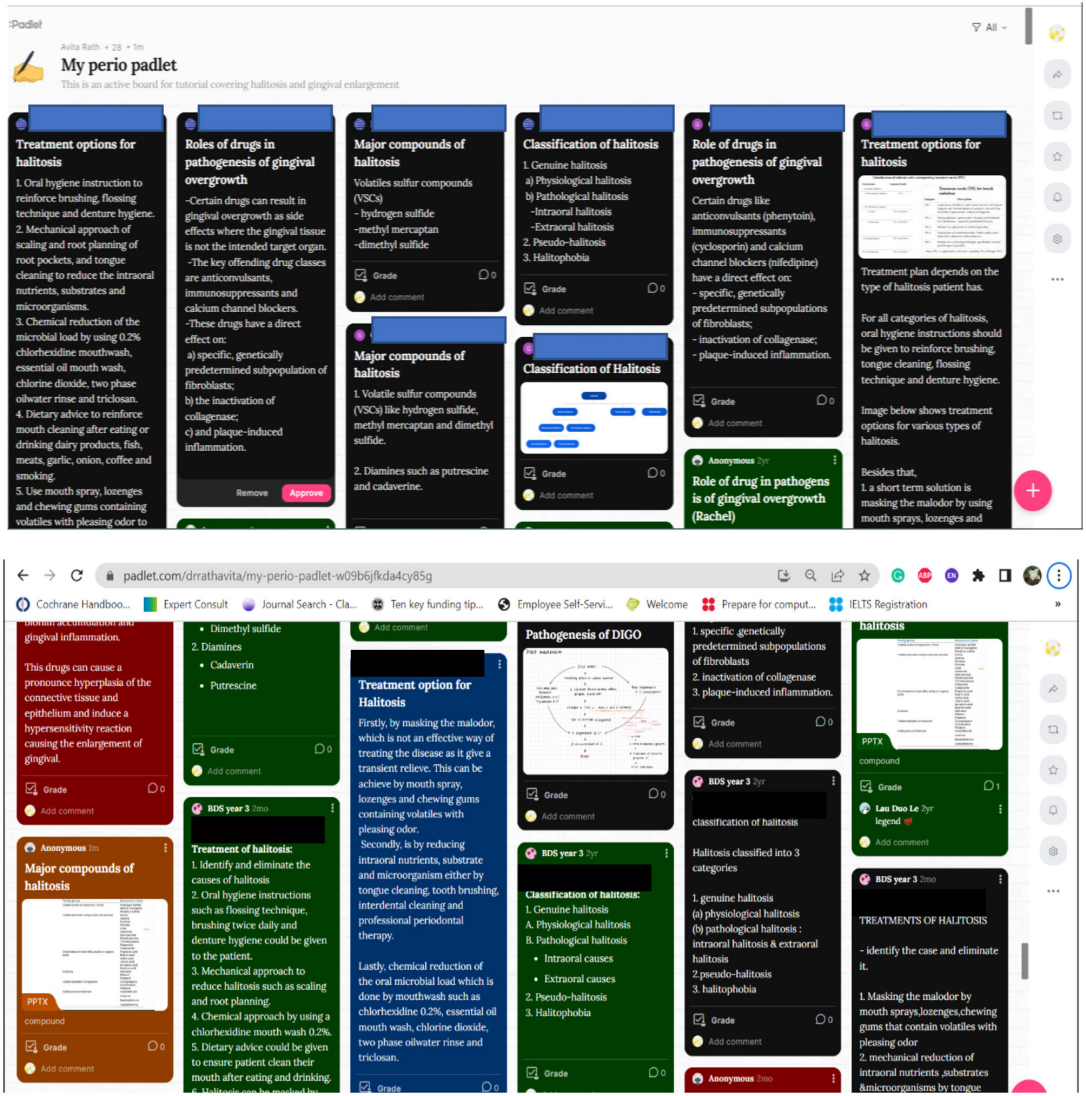
Padlet-based peer feedback activity by Year 3 dental undergraduate students.

Padlet was used for a week-long formative peer feedback activity. Students received clear instructions and exemplars from the previous cohort to guide their participation. The exemplars were provided at the beginning of the activity to clarify expectations and provide a model for quality contributions, emphasizing that these were guides and not templates. Students were explicitly instructed to use them as guides, not templates, and to express their unique perspectives. Students were encouraged to post answers, comments, and feedback using various multimedia formats and to comment on at least two posts from their peers (Figure 1). The use of exemplars aimed to clarify expectations and provide a model for quality contributions. While exemplars can potentially influence student thinking, they were carefully selected to represent diverse approaches and stimulate original thought.

### Data collection

Data were collected through the following methods:

- Padlet posts: all student posts, comments, and feedback were archived and analyzed.
- Student reflections: at the end of the activity, students were asked to reflect on their learning experiences and the impact of Padlet on their understanding of the topics through written reflections.

### Data analysis

I used Braun and Clarke's (22) reflexive thematic analysis to identify and interpret patterns and themes in the collected data. The analysis followed six structured steps:

- Familiarization with the data: I immersed myself in the data by repeatedly reading the Padlet posts and student reflections to gain a comprehensive understanding.
- Generating initial codes: I systematically coded the data, highlighting features that were relevant to the research questions. Codes were developed inductively, focusing on recurring ideas and significant insights.
- Searching for themes: the initial codes were grouped into potential themes, reflecting common patterns and meanings across the dataset.
- Reviewing themes: I refined and reviewed the themes to ensure they accurately represented the data, checking for coherence within themes and distinctiveness between themes.
- Defining and naming themes: each theme was clearly defined and named to encapsulate its essence and convey the overall story of the data.
- Producing the report: I constructed a detailed narrative of each theme, supported by illustrative quotes to provide evidence and strengthen the validity of the findings.

The analysis focused on student perceptions of collaborative learning, feedback literacy, the implementation of Nicol and MacFarlane's feedback principles (4), and the role of trust in these processes. Nicol and MacFarlane's feedback principles provided a framework to interpret how students engaged with feedback and self-assessment and how Padlet facilitated dialogue and reflection.

### Reflexivity

Reflexivity was a critical component of the analysis. As both a researcher and an educator, I was aware of the potential power dynamics at play, given that I was assessing my students. Power relations can influence how participants respond, especially in educational contexts where the researcher holds a position of authority (24). Several measures were taken to mitigate the influence of these power relationships on the study. First, participation in the research was entirely voluntary, and students were assured that their decision to participate or the nature of their responses would not impact their grades or standing in the course. Second, I engaged in continuous self-reflection throughout the research process, journaling my thoughts and biases to remain aware of how my position of authority could influence data interpretation.

I also created a psychologically safe environment for students by emphasizing the anonymous nature of the Padlet activity. This anonymity was intended to reduce the pressure students might feel to conform to perceived expectations and to promote honest and authentic engagement. Despite these measures, my role may have subtly influenced students' willingness to participate or how they expressed themselves. To address this, I actively sought to consider multiple interpretations of the data and engaged in discussions with colleagues to challenge and refine my analysis.

## Results

The thematic analysis of Padlet posts and student reflections revealed three main themes: the power of asynchronous communication, the importance of trust and psychological safety, and the development of cognitive flexibility. These themes illustrate how Padlet facilitated collaborative learning and feedback literacy among dental students.

### The power of asynchronous communication

The asynchronous nature of Padlet allowed students to reflect and respond at their own pace. This facilitated richer and more thoughtful dialogues among peers. One student noted, “*The asynchronous nature of the platform allowed us to really delve into the topics and learn from each other's insights.”* Another student shared, “*I found myself questioning my own understanding and expanding my knowledge as I read my peers' posts.”* Students also built on each other's contributions, sharing diverse perspectives and reaching a shared understanding of complex topics. The platform enabled students to engage deeply and collaboratively, creating a sense of community (13).

### Trust and psychological safety

Padlet's anonymity feature created a safe environment for open communication. Students felt more comfortable sharing ideas without fear of judgment. One student remarked, “*I felt more comfortable giving and receiving feedback on Padlet because I did not have to worry about being judged.”* However, some students expressed concerns about the potential for anonymity to reduce accountability, highlighting a need for balance in the use of this feature. Overall, the sense of psychological safety encouraged more honest and constructive feedback among peers (18).

### Cognitive flexibility

Engaging in asynchronous discussions provided students with opportunities to consider multiple perspectives and develop more nuanced understandings. Students demonstrated cognitive flexibility by crafting thoughtful responses and engaging with their peers' ideas. One student mentioned, “*Having time to think about feedback before responding made a big difference in how I understood and used it.”* This flexibility in thinking allowed students to reflect critically on their learning and adjust their understanding based on peer input (23).

## Discussion

This study provides valuable insights into the potential of Padlet for enhancing collaborative learning and feedback literacy in dental education. As the demands for more flexible and engaging learning environments increase, the integration of digital platforms into educational practice is becoming crucial. The findings from this study illustrate how tools such as Padlet can transform traditional feedback practices by fostering meaningful engagement and reflective learning.

The asynchronous nature of Padlet emerged as a pivotal feature in promoting cognitive engagement. Unlike synchronous communication, which often demands immediate responses, asynchronous platforms allow students to reflect deeply on their peers' contributions. This supports the CoI framework, emphasizing the role of cognitive presence in creating meaningful learning experiences (13). The ability to engage thoughtfully enhances self-regulated learning, which is crucial in professional education, where continuous reflection and critical thinking are required. Recent research by Mehta et al. found that Padlet facilitates open communication and peer learning across disciplines, although students' perceptions may vary depending on their field of study (5). Similarly, Naamati-Schneider and Alt demonstrated Padlet's versatility in health management education, using it for live sessions, pre-session activities, and post-session reflections to promote engagement and deep learning (23).

The theme of psychological safety, facilitated by Padlet's anonymity feature, was another critical finding. Psychological safety, as defined by Edmondson, is essential in environments where individuals feel secure sharing ideas without fear of negative consequences (19). In this study, students reported that anonymity allowed for more honest and constructive feedback, consistent with Carless and Boud's emphasis on trust as foundational for effective feedback practices (3). An empirical review by Panadero and Alqassab demonstrated that anonymity in peer feedback increases students' willingness to provide candid critiques (25). However, the challenge of maintaining accountability in anonymous settings remains. Jong et al. emphasized that while anonymity can foster collaboration and openness, clear guidelines are necessary to prevent misuse and ensure constructive interactions (21).

**Figure 2:**
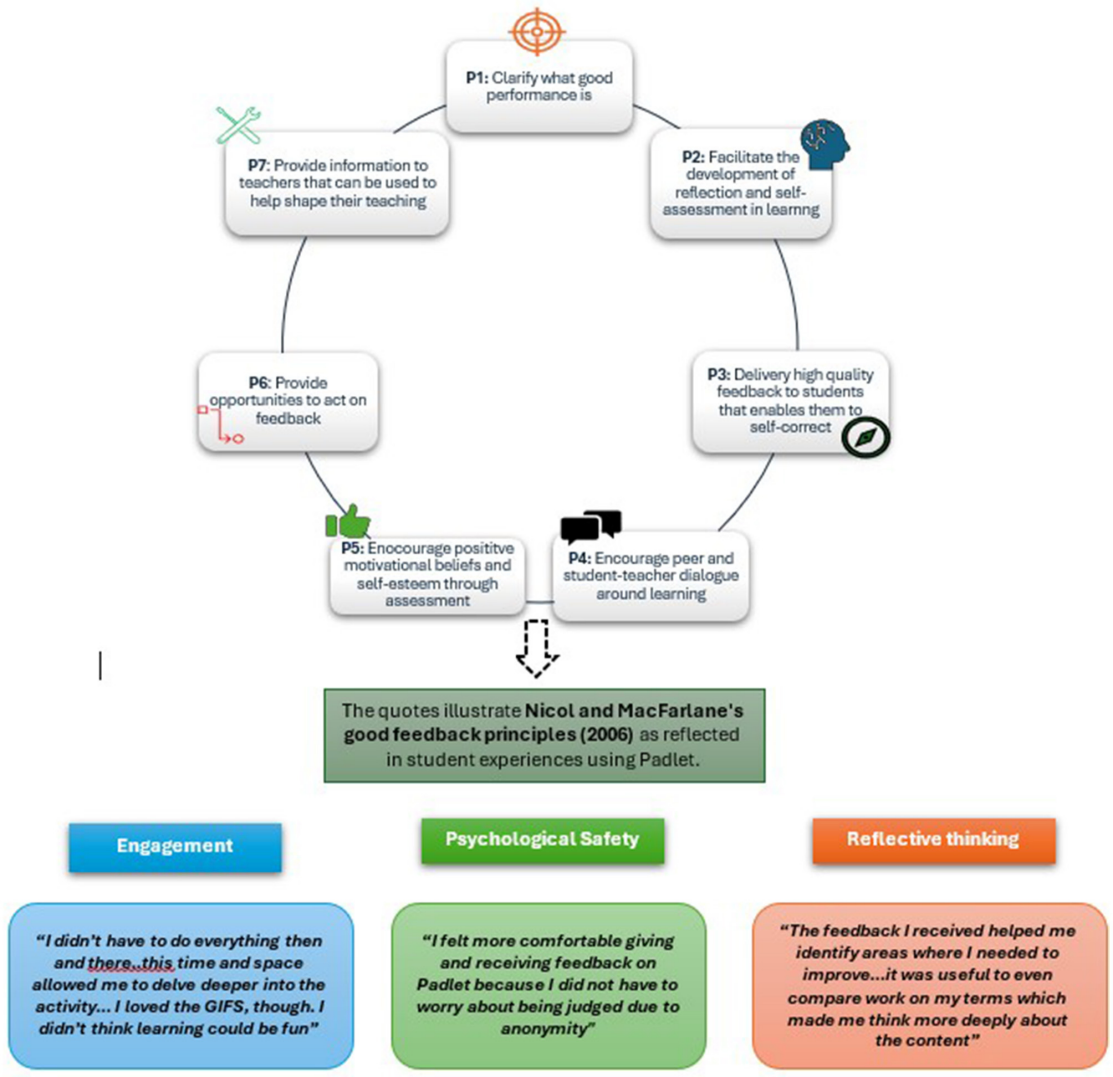
Overview of Padlet-based peer feedback interactions. The colored boxes contain direct student quotes, illustrating how Nicol and MacFarlane's feedback principles were applied during the activity. Each quote represents key themes such as engagement, psychological safety, and reflective learning.

Cognitive flexibility, as facilitated by Padlet, is another essential outcome identified in this study. The ability to engage with diverse perspectives and critically reflect on one's own understanding is fundamental in developing adaptive expertise, particularly in fields such as dentistry. This finding aligns with transformative learning theory, which emphasizes the importance of critical reflection in enabling learners to challenge and transform their perspectives (7). The asynchronous format allowed students to reconsider their viewpoints and engage in meaningful dialogue. It supports Laurillard's (8) concept of dialogic learning, which stresses the importance of interaction and reflection in knowledge construction. Additionally, Slavin's study on cooperative learning highlights the significance of structured collaborative environments in enhancing engagement and learning outcomes, a principle Padlet's design naturally supports by facilitating student-to-student interactions (28). By creating a space for sustained reflection and thoughtful engagement, Padlet supports the development of cognitive flexibility, a skill increasingly valued in professional practice. As shown in Figure 2, the application of Nicol and MacFarlane's feedback principles facilitated cognitive flexibility, psychological safety, and deeper engagement through reflective thinking.

These findings highlight the transformative potential of digital platforms in education, but they also underscore the importance of thoughtful implementation. Tools such as Padlet must be integrated into well-designed pedagogical strategies that emphasize active learning, reflection, and meaningful interaction. As Bennett et al. (29) noted, the success of digital tools depends heavily on the overall design and facilitation of learning activities. Educators must carefully balance the benefits of flexibility and psychological safety with measures that ensure accountability and productive discourse.

### Recommendations for educators

To maximize the benefits of using Padlet in educational settings, the following recommendations have been developed based on the findings of this study:

- *Facilitate reflective learning:* design activities that encourage sustained, thoughtful engagement. Providing students with sufficient time to reflect on and respond to prompts can promote deeper learning and cognitive presence.
- *Foster psychological safety:* use anonymity features to create a safe environment for honest feedback. However, establish clear guidelines to balance openness with accountability, ensuring that interactions remain respectful and constructive.
- *Promote cognitive flexibility:* incorporate structured prompts that require students to consider multiple perspectives and reflect critically on their understanding. This approach can help develop adaptability and critical thinking skills, which are essential in professional fields such as dentistry.
- *Provide scaffolding and support:* offer examples of high-quality feedback and clear instructions to guide student interactions. Scaffolding can help students engage constructively and understand the expectations for meaningful participation.
- *Monitor and manage online discussions:* actively facilitate discussions to ensure they remain focused and productive. Setting norms for communication and providing timely interventions when necessary, can help maintain a positive learning environment.

### Limitations and future research

This study has several limitations. The research was conducted at a single institution with a relatively small sample size, which may limit the generalizability of the findings. Additionally, the reliance on self-reported data introduces the possibility of response bias. Future research should explore the impact of digital platforms such as Padlet across diverse educational contexts and examine long-term outcomes on student learning and feedback literacy. Comparative studies investigating features such as anonymity, multimedia capabilities, and their effects on student engagement and learning would provide a more nuanced understanding of the pedagogical value of these tools.

## Conclusion

This study highlights the potential of Padlet in enhancing collaborative learning and feedback literacy among undergraduate dental students. The asynchronous and anonymous features of Padlet contribute to a learning environment that supports reflective thinking, trust, and cognitive flexibility. These unique features make Padlet a valuable tool for fostering student-centered learning and self-regulation in dental education. The practical insights from this study emphasize the importance of thoughtfully designed pedagogical strategies that leverage digital tools to create engaging and trusting environments, ultimately promoting collaboration, effective feedback, and student success.

## Data Availability

The raw data supporting the conclusions of this article will be made available by the author, with reservation to protect anonymity and confidentiality of participants.
